# In Vitro Characteristics of Phages to Guide ‘Real Life’ Phage Therapy Suitability

**DOI:** 10.3390/v10040163

**Published:** 2018-03-30

**Authors:** Eoghan Casey, Douwe van Sinderen, Jennifer Mahony

**Affiliations:** School of Microbiology and APC Microbiome Ireland, University College Cork, T12 YT20 Cork, Ireland; eoghan.casey@ucc.ie (E.C.); j.mahony@ucc.ie (J.M.)

**Keywords:** pH stability, phage-host interactions, genomics, antibiotic-resistance, phage preparation, lysins, biofilms

## Abstract

The increasing problem of antibiotic-resistant pathogens has put enormous pressure on healthcare providers to reduce the application of antibiotics and to identify alternative therapies. Phages represent such an alternative with significant application potential, either on their own or in combination with antibiotics to enhance the effectiveness of traditional therapies. However, while phage therapy may offer exciting therapeutic opportunities, its evaluation for safe and appropriate use in humans needs to be guided initially by reliable and appropriate assessment techniques at the laboratory level. Here, we review the process of phage isolation and the application of individual pathogens or reference collections for the development of specific or “off-the-shelf” preparations. Furthermore, we evaluate current characterization approaches to assess the in vitro therapeutic potential of a phage including its spectrum of activity, genome characteristics, storage and administration requirements and effectiveness against biofilms. Lytic characteristics and the ability to overcome anti-phage systems are also covered. These attributes direct phage selection for their ultimate application as antimicrobial agents. We also discuss current pitfalls in this research area and propose that priority should be given to unify current phage characterization approaches.

## 1. Introduction

Frederick Twort and Felix d’Hérelle are accredited as the founding fathers of phage biology having recognized that bacteriophages or “bacteria eaters” are present wherever bacteria are found [1,2]. During World War I, the unsanitary conditions in the trenches on the battle-fronts caused infections such as dysentery affecting numerous soldiers. Felix d’Hérelle studied the bacterial cause of these infections and identified *Shigella* as the etiologic agent of this rampaging infectious disease. Frederick Twort demonstrated the existence of a propagatable bactericidal agent in 1915, while independently (or not) Felix d’Hérelle co-incubated fecal filtrates with the isolated bacteria in petri dishes resulting in the killing of the bacteria (d’Hérelle’s prior knowledge of Twort’s research remains uncertain, reviewed here [3]). The first description of the co-existence and relationship between bacteria and their infecting phages was a historic one that created the opportunity to develop treatments against bacterial infections for which therapies were not available at that time. During the 1920’s and 1930’s d’Hérelle blazed the trail of phage therapy by successfully developing phage-based treatments against a range of human infections including those caused by *Shigella dysenteriae*, *Salmonella typhi*, *Escherichia coli*, *Pasteurella multocida*, and *Vibrio cholerae*.

The discovery of mold-derived penicillin production (by *Penicillium notatum*) by Alexander Fleming in 1928 and the subsequent discovery of its broad-spectrum of antibacterial activity and relative ease of large scale production delivered a major blow to developments in phage therapy. The need for rapid production of agents that could effectively treat wound infections during World War II accelerated the rise of antibiotic-based therapies, which were highly effective and were considered a wonder drug at that time. However, little was known about the modes of action of either phages or antibiotics, and phage therapy research was almost completely abandoned in favor of further development of the antibiotic industry. The lack of adequate controls in these early phage experiments combined with the lack of clarity regarding phage efficacy were among the reasons that antibiotic research and development out-paced that of phage therapy research. While antibiotics were successfully exploited for the ensuing decades, the rapid rise of antibiotic resistance and the emergence of so-called “superbugs”, such as methicillin-resistant *Staphylococcus aureus* (MRSA), carbapenem-resistant *Acinetobacter baumannii* and *Enterobacteriaceae* and *Clostridium difficile*, among others, are crippling to modern healthcare facilities. Furthermore, the World Health Organization (WHO) has stated that alternatives to antibiotics are required to treat such superbugs, which are estimated to cause 25,000 deaths in Europe and 23,000 deaths in the U.S.A. each year (WHO statement, February 2017). Plasmid-encoded antibiotic resistance represents a particularly challenging threat to society due to possible transfer or mobilization of such extra-chromosomal elements between bacterial strains or even species. Therefore, phage therapy or phage-based treatments may provide a solution to this pressing health issue in modern society, just over a century since their initial identification.

Both antibiotics and phages have inherent associated risks, draw-backs, and benefits. Phage therapy presents the possibility of applying a more targeted, narrow spectrum treatment, thereby providing greater specificity than most antibiotics. Furthermore, phages may be delivered as intact phage particles/phage cocktails, while phage-derived proteins may also be produced to overcome many of the concerns associated with delivering intact phage particles into humans. Considerable work has been undertaken in recent years to isolate and characterize phages with therapeutic potential against a range of pathogenic bacteria. While many phage/phage cocktails have proven effective in lab-scale trials, this has not always been matched by successful in vivo trials, cautioning against too much optimism with regards to the success of phage therapy when applied in real life settings. Careful evaluation of the in vitro phenotypes of phages is therefore required before they may be considered suitable for clinical trials for the purpose of animal or human therapy [4,5]. In this review, we assess the methods by which phages are isolated and characterized for therapeutic consideration, the benefits and pitfalls of current approaches, while also considering alternative approaches for the future. Furthermore, we explore the advantages and disadvantages of phage cocktails and phage-derived products, and the most recent advances in combination therapies as a 21st century approach to deal with bacterial infections.

## 2. Phage Isolation

As bacteriophages represent the most abundant biological entity in the biosphere [6], isolation of phages infecting pathogenic strains of interest should not represent a significant hurdle. Isolation of phages against pathogenic species requires identification of areas where the pathogenic host is abundant. In particular, sewage samples represent a plentiful source of phages for use in phage therapy due to the presence of many human pathogens [7]; however, any environment where the pathogenic host is present represents a potential source of phages. After identification of these environments, methods can be employed to isolate phages of particular value in phage therapy.

In general, the utilization of a diverse range of strains as isolating hosts should lead to the isolation of a diverse range of infecting bacteriophages. The use of reference collections as isolation hosts for pathogens of interest should be the gold standard for phage isolation studies. Collections such as the ECOR collection of *E. coli* [8] and the *Salmonella* SARA and SARB collections [9,10], purported to represent the full intra-species diversity have been used in phage isolation studies and can be helpful in generating an arsenal of phages with a high likelihood of infecting strains of clinical relevance with respect to the targeted species (as such phages are presumed to recognize any of the diverse range of cell surface receptors that may be present among such strains) [11]. Even more preferable are studies utilizing reference collections in addition to strains of clinical relevance, leading to the generation of highly diverse phage collections [12]. A further development in this area is the SBS (step-by-step) method, which can be applied when attempting to isolate a phage against single strains, and which was first developed for *Klebsiella pneumoniae*. This involves isolation of phages against the strain of interest in addition to bacteriophage-insensitive mutants of the same strain. In short, a phage is isolated against a strain of interest, then a resistant variant of this strain is generated through exposure to the isolated phage. Following this, a new phage is isolated against this resistant variant and so on until the latest phage-resistant variant is sensitive to the original phage. The mechanisms through which host resistance is achieved were not investigated, although presumably it relies on efficient CRISPR (Clustered Regularly Interspaced Short Palindromic Repeats) spacer incorporation. In essence, this method will generate a cocktail of phages infecting both the original bacterium and many phage-resistant variants, reducing the possibility of variants emerging which are resistant to the cocktail during application [13]. Furthermore, it has been theorized that standard bacteriophage isolation and enrichment protocols involving single strains are biased against broad range bacteriophages, (unintentionally) selecting for those with most avid adsorption to host cells. The use of a mixed host enrichment procedure has previously led to the isolation of broad host range bacteriophages, which arguably are the most valuable candidates for phage therapy [14].

At the isolation step, care should be taken to ensure that only virulent phages are selected as candidates for phage therapy. It is advised that phages capable of lysogenic conversion be avoided, as these will easily convert hosts into (phage-resistant) lysogens, thus making them incapable of causing immediate lysis [15]. Avoidance of plaques with turbidity, typical of lysogenic phages, will assist in the selection of lytic phages, but as some temperate phages produce clear plaques, further methods are required for lifestyle validation [11]. With the rapid advancement of genome sequencing technologies, it is now feasible to sequence the genomes of all candidates, therefore allowing for more definitive exclusion of phages that encode integrases, site-specific recombinases, and repressors of the lytic cycle [7]. Indeed, a genomic approach to guide phage selection has in recent years gained in popularity and is covered in more detail below.

Long-term storage and stability of selected phages is desirable, and a good candidate for phage therapy should be one that maintains infective ability upon storage. Several methods have been proposed for maintenance of phage stocks including storage at 4 °C [16], storage at −80 °C with the addition of glycerol [17], and lyophilization of phage stocks, with the latter proposed to be a particularly effective method for long term storage [18]. A novel method involving the storage of phages as injected DNA in freshly infected frozen cells has shown promise, although some strains were shown to be poor storage hosts [17]. It has been noted that due to variation amongst bacteriophages a “gold-standard” phage storage condition remains to be identified, and long-term storage of phages is likely to be phage dependent [19]. Ackermann et al. have detailed the stability and instability of various phages, and for example described that *Bacillus* phage CP-54Ber suffers a 7-log reduction in infective ability following three months of storage at 4 °C (representing the most common phage storage temperature), exemplifying this diversity [16]. Therefore, in vitro assays to determine optimum storage conditions for each phage of interest should be undertaken [20].

## 3. Characterization of Phages for Phage Therapy Applications

### 3.1. Genomic and Morphological Characteristics

A lack of uniformity in the approaches to assess phage isolates in the laboratory for their suitability as a therapeutic agent is possibly one of the biggest stumbling blocks to phage therapy. Recent phage therapy studies that report genome characterization are typically partnered with morphological analysis, which is a routine analysis step. While the isolation of phages has been a continuous and ongoing effort of phage biologists globally [21,22,23,24,25], it should be noted that considerable collections of phages that have the potential for therapeutic trialing and application, exist. One such collection of nine *E. coli* phages has recently been assessed to identify the common microbiological and genomic characteristics of phage isolates exhibiting therapeutic potential in vivo [26]. This historic collection of *E. coli* O18:K1:H7 phages was characterized as being comprised of six *Podoviridae* phages and three *Siphoviridae* phages. Four of the podophages exhibited the most significant host-infecting efficacy among the collection, and genomic analysis revealed that these isolates were all members of the same species bearing at least 99% identity to each other. The presence of a DNA-dependent RNA polymerase was present only in the genomes of “fast replicating” phages. This protein is purported to cause a shutdown of host transcription (5 min after infection), resulting in a much shorter latent period than non-RNA polymerase-encoding phages. This confers a more efficient replication cycle on the phages, a property suggested to underpin the success of these isolates in in vivo trials. Indeed, the relatively simple structures associated with the podophage may lend itself to shorter latent times and larger burst sizes compared to phages that possess elaborate and decorative structures. In contrast, two recently isolated, *Clostridium difficile*-infecting *Myoviridae* phages were shown to exhibit a broad host range, infecting strains from approximately half of the strains that represent the 20 ribotypes of *C. difficile* [24]. The more extensive host range of these isolates relative to other *Myo-* and *Siphoviridae* phages from the same study highlights that large phages such as myophages may have therapeutic potential, while the infective success of various phage morphologies appears to be host-specific. Therefore, identification of genetic and morphological features associated with highly infective phages may represent useful markers to guide the selection of phages for subsequent therapeutic application. However, these markers may be genus- or species-specific, and therefore caution should be taken not to make generic recommendations for the identification process of phages with therapeutic potential.

Notwithstanding the above-mentioned limitations, genome sequencing permits the rapid identification of undesirable features that would quickly “rule out” unsuitable phages. For example, as mentioned in Section 2, lysogenic phages are generally not considered suitable for phage therapy, since their integration into the host genome may confer alterations on the host phenotype (lysogenic conversion). Additionally, in the integrated state, they may recombine with genetic elements of other (pro)phage or bacterial genomes and acquire undesirable features such as pathogenicity islands or antibiotic resistance markers, among others. Genome characterization permits the identification of lysogeny-associated functions including repressors and integrases, which are often readily identifiable. Furthermore, the presence of recombination-related functions in phage genomes may present issues in terms of genetic instability. Analysis of genome stability is an area that has not yet been deeply investigated, although recent advances in protein function prediction tools such as Pfam [27] and HHpred [28] have significantly improved the quality and accuracy of genome annotations, which may contribute to the development of this aspect.

Morphological assessment by electron microscopy is a standard characterization step of almost all newly reported isolated phages. Although this requires specialized equipment and expertise, it is an essential characterization step, and undoubtedly will continue to be the cornerstone of phage characterizations. Electron microscopy may be particularly useful in assessing the stability of stored phage isolates. Relying on microbiological assays such as the standard plaque assay for phage enumeration may not reveal the true extent of the stability of the produced phages, whereas electron microscopy may reveal the presence of additional “ghost” or degraded particles.

### 3.2. Host Receptor Identification

One of the concerns associated with human phage therapy is the emergence of phage-resistant variants of pathogenic bacteria with increased fitness. The host range of a phage reflects its ability to (lytically) infect strains within a given test panel where narrow host range phages infect a small number of strains and broad host range phages infect a wide range of strains. Phages may exhibit narrow or broad host range depending on (i) the presence of anti-phage mechanisms in the test strains and; (ii) the presence of generalized (highly conserved) or specialized (variable, non-conserved) host-encoded phage receptors. While broad host range phages are generally more acceptable due to the increased likelihood that clinical isolates that emerge will be infected, narrow host range phages may be useful in certain scenarios. In contrast to antibiotics, which are broad spectrum antimicrobial agents, the use of narrow host range phages presents a new opportunity. It allows the possibility of isolating phages against prevalent and specific strains of pathogenic bacteria and delivering a treatment without the problem of host dysbiosis. The identification of narrow spectrum phages combined with the SBS approach (mentioned in Section 2) could reduce the risk of the phages becoming defunct upon the emergence of resistant variants of the target strains and present a useful approach to developing next generation therapies. However, as with all treatments, narrow host range phages have their limitations. As pathogens evolve and populations diversify, it is necessary to have monitoring programmes to continually evaluate prevalent strains of problematic pathogens. Therefore, corresponding phage screening programmes should be initiated against pathogenic strain collections including the most recent clinical isolates on a continuous basis. This would require a concerted approach by various phage research groups and associated funding from government and other agencies to ensure the “future-proofing” of this approach. The peak-and-trough profile of phage research over the past century indicates that this is difficult, however, with the growing demand for alternative antimicrobial therapies, it is expected to gain support, at least in the foreseeable future.

While targeted narrow host range phage therapies may have potential for specific applications, it is likely that broad host range phages will continue to be the preferred option as they possess a more powerful destructive potential against a wider range of pathogenic isolates. Furthermore, even those phages that are classified as exhibiting a broad host range would still be considered to possess a narrow activity spectrum relative to antibiotics. Antibiotics may be effective against multiple genera of bacteria, while phages are rarely genus-specific, but mostly species- or strain-specific. During the past two decades, interactions between phages and their host bacteria have become the subject of intense research scrutiny [29,30,31,32,33,34,35,36,37]. The availability of bacterial and phage genome sequences has facilitated studies in which phage-resistant derivatives of bacterial host strains are sequenced to uncover the genetic basis of phage-resistance and by inference the receptors that phages recognize [38,39]. Conversely, the isolation of phage mutants that can circumvent such phage-resistant derivatives have been characterized to understand the molecular basis of phage infection and the genes that encode host recognition functions [40,41]. Through such analyses, the interactions of a wide range of phages and their bacterial hosts are now well defined and in theory the methods can be adapted to study any phage-host combination. To alleviate concerns regarding the emergence of phage-resistant variants of pathogenic bacterial strains, phage cocktails incorporating phages that employ different receptors is advisable. However, this requires knowledge of the receptor types employed by the incorporated phages. For this reason, phage-host interaction studies are vital to the design of robust phage cocktails.

Phage-host interactions require both a host-encoded receptor(s) and a phage-encoded receptor binding protein (RBP). The receptor presented on the cell surface may be a carbohydrate, protein or (lipo-)teichoic acid moiety, or a combination of these. The fundamental differences in the composition of Gram-negative and Gram-positive cell walls dictate the types and range of interactions that may occur between phages and their respective hosts. For example, the majority of Gram-positive bacteria-infecting phages are reported to recognize saccharidic moieties on the cell surface, while those of Gram negative-infecting phages such as those infecting *E. coli* and *Salmonella* are less biased, with several coliphages known to recognize proteinaceous and saccharidic receptors according to a recent review of phage receptors [42]. This extensive review of phage receptors has culminated in the generation of the Phage Receptor Database (PhReD, https://phred.herokuapp.com/), which is a highly informative resource for phage biologists.

For many phages, a single receptor is required for the recognition and attachment stages of the infection process while for others, two (or more) components are required. Coliphages are undoubtedly the best studied Gram-negative-infecting group of phages with model *Myoviridae* and *Siphoviridae* phages such as T4 and lambda, respectively, representing paradigms of infection of this bacterial species (For reviews, see [43,44]). Lambda, with its long non-contractile tail, is an example of a phage that requires a single receptor, i.e., the protein LamB [29], while the long tail fibers of the myophage T4 bind reversibly to the protein OmpC before the short tail fibers commit phage binding to the heptose moiety of the lipopolysaccharide (LPS) [45,46]. In the review of phage receptors mentioned above, 26 coliphage interactions were covered, of which eight require both cell envelope-associated proteins and sugar moieties, while ten and eight coliphages, respectively, require proteins or carbohydrate moieties alone for host adsorption [42]. A similar analysis of currently characterized *Salmonella* phages tells us that 11 phages employ proteinaceous receptors, seven recognize carbohydrate moieties on the cell surface, while one requires both moieties to adsorb to their host. In contrast, all *Pseudomonas* phage-host systems characterized to date attach to saccharidic receptors. Therefore, it is clear that there is a diverse array of interactions at play among these phage-host combinations.

In contrast, the interactions of the majority of characterized Gram positive-infecting phages involve saccharidic molecules [42,47]. Among the model phage-host systems of Gram-positive bacteria are those of *Bacillus subtilis* and its infecting phage SPP1 [31,35] and those of the dairy bacterium *Lactococcus lactis* and members of the 936 and P335 phage groups [34,48,49]. While these phage-host interactions bear no direct clinical relevance, studies of these two Gram-positive hosts and their phages have consolidated studies of the interactions of clinically relevant Gram-positive hosts such as *Listeria monocytogenes*, *Bacillus anthracis* and *Staphylococcus aureus*, among others. The level of structural detail that now exists for SPP1, and the 936 and P335 lactococcal phages have provided insights into the nature and interactions of phages of other Gram-positive bacteria and those with clinical relevance. Module shuffling to accommodate host interaction flexibility is not a new concept; however, the structural characterization of phages and phage components (particularly the distal tail components including the host recognition devices) have generated data pertaining to modules that may be present and conserved among phages of different bacterial genera. An example of this is the so-called “evolved” distal tail protein (Dit) of phages of *Lactobacillus* and *Lactococcus* [48,50], in which a carbohydrate-binding domain (CBD) is inserted to expand/enhance the binding capabilities of the carrying phage. Furthermore, another CBD was structurally characterized in the accessory baseplate (distal tail appendage) protein (BppA) of the lactococcal phage Tuc2009 [33] and based on HHpred analysis has been identified in a number of other lactococcal, *Lactobacillus* and streptococcal phage proteins, highlighting that this domain is found in phages infecting other bacterial genera [48,50]. Taken together, knowledge on the diversity of interaction types will guide the selection of phages for inclusion as therapeutic agents either alone or as part of a cocktail. 

### 3.3. Stable Storage, Administration and Effectiveness in Trials

In order to be acceptable for therapeutic application, a phage must retain its infectivity under storage conditions over extended periods of time, and be able to withstand the administration process and route. To evaluate this, phages are often tested for robustness to pH treatments and simulated gastric juices to mimic the oral route of administration [51,52]. Additionally, other studies have evaluated the stability of phages upon freeze drying, spray drying and cold storage to define their appropriateness for the clinical setting [53,54]. Such studies have demonstrated that the stability is pH-, excipient- and phage-dependent. Therefore, it is essential that each candidate phage is assessed using a range of conditions to define the appropriate preservation/storage conditions. In addition to preserving the phage, the cultivation medium, scale up and harvesting of phage preparations are all aspects that require careful scrutiny in order to ensure that the phage is capable of transitioning from the laboratory to the clinic. A recent study examined such characteristics of T4-like coliphages [55]. Here, it was demonstrated that the employed growth medium had minimal impact on the phage titers achieved, while growth phase (i.e., early exponentially growing cells were more effective than lag or late exponential phase cells) and the choice of host strain were important factors in producing a high phage titer. The authors also evaluated different propagation approaches including 2 L Erlenmeyer flasks, 16 L stirred fermentation tanks and 10 L wave bags, as well as different methods of purification including ultrafiltration, chromatography and ultracentrifugation. Phages were shown to exhibit flexibility to scaling up and purification, permitting inexpensive and relatively fast production of lysates of coliphages for application. Since such therapies are also increasingly aimed at providing cost-effective and practical solutions to commonplace infections in developing countries, the propagation and purification techniques need to be considered as specialized laboratory equipment such as ultracentrifuges may not be readily available [55].

The phage (or phage-derived product) administration route also dictates the type of characteristics that are required. For example, if the phage product is to be administered orally to treat enteric infections, the phage should be able to withstand low pH, gastric conditions and/or be suitable for encapsulation for delivery to the desired site. Encapsulation trials have been undertaken with phages of various pathogens including *C. difficile* and enterohemorrhagic *E. coli* using a range of materials including Eudragit^®^ S100 (a pH responsive polymer), and in the presence of alginate or pectin as base polymers, among others [56,57]. Such microencapsulation was shown to retain infection efficacy of the tested phages upon exposure to simulated gastric conditions and the associated acidity. For treatment of respiratory infections and chronic conditions, an aerosol-based application may be the preferred route of administration requiring that the phage should retain infectivity following freeze- or spray-drying and administration [58].

A phage’s potential for application is commonly based on animal trials prior to consideration of human trials. Animal trials most often involve a murine model system although larger animals have been employed in the assessment of phages destined for the treatment of animals in the food chain [59]. Trials in sheep and cattle inoculated with *E. coli* O157:H7 highlighted the need for adequate controls as endemic phages contributed to the phage population that was shed, interfering with the results of the therapeutic analysis. Animal trial-based assessment of phage therapy applications is a contentious issue and must be limited where possible. Some recent studies have performed preliminary trials using wax moth (*Galleria mellonella*) larvae in order to allay such ethical concerns. The *Galleria* larval model is a useful system since these organisms possess complex innate immune systems, and, additionally, similarities have been identified between insect larval epithelial cells and intestinal mammalian cells [60]. This model system is inexpensive and requires little specialist training, and is therefore useful to define which phages display genuine therapeutic potential and are suitable for further testing.

Relatively few studies have investigated the interactions between bacteriophages and the immune system, however, persistence in the face of the host immune system represents an essential characteristic of candidates for phage therapy. It is known that phages are immunogenic, with studies of T4 coliphage showing stimulation of antibody production through both oral and subcutaneous injection with significantly lower immunogenicity observed via the oral administration route [61]. However, persistent injection at a dose higher than what would normally be administered was required to elicit a marked immune response [61]. Continual exposure of the body to the intra-body phageome presumably has a role to play in this, and it has been suggested that the apparent lack of immune response is due to chronic phage exposure during evolution [62]. Indeed, phage exposure causes an immunomodulatory response, displaying an inhibitory effect of T-cell proliferation and downregulation of antibody production contributing towards the homeostasis of the immune system [63,64].

While phages and phage cocktails present an opportunity to combat the current shortage of antimicrobial therapies, phage therapy remains an unattractive option to many regulatory bodies. However, advances in phage biology, genome sequencing and molecular biology may provide the knowledge to overcome such regulatory concerns through the exploitation of phage-derived proteins as we will discuss below.

## 4. Phage Particles Versus Phage-Derived Products

Endolysins are produced by phages at the end of their infection cycle in order to release progeny phages from the host cell. They are characterized by their ability to hydrolyze the peptidoglycan layer of the bacterial cell wall [65]. Endolysins represent the most promising lytic enzymes used in phage therapy, possessing several advantages over other candidates. They are effective immediately (in contrast to the lag time exhibited by bacteriophage particles), lysing target cells within seconds of first contact. For example, a streptococcal lysin specific for groups A, C and E was shown to completely sterilize a culture containing 10^7^ colony forming units of the group A *Streptococcus* strain D471 in vitro. This in vitro activity was a good indicator of in vivo effectivity as mice which had been heavily colonized in the upper respiratory tract with a streptomycin-resistant group A *Streptococcus* (T14/46) showed complete eradication of said strain two hours after administration of purified lysin [66]. A further advantage of endolysin treatment is that, although purified enzymes are antigenic, they are capable of avoiding the humoral immune response in a study of endolysin application to methicillin resistant *S. aureus* [67]. Here, endolysin LysGh15 administration was shown to elicit specific-IgG antibody production, although incubation of LysGh15 with anti-LysGH15 serum showed no difference in bactericidal activity when compared to LysGH15 incubated with normal mouse serum. This indicates that purified lysins are suitable for application without inactivated by the host immune system. Endolysins also display a higher target specificity compared to antibiotics (thereby reducing the potential for resistance development). Purportedly, no resistance to endolysin activity has yet been observed due to the evolutionary link between phage endolysin and host cell autolysin [68]. A significant disadvantage associated with the use of purified endolysins is the lack of efficacy against most Gram-negative pathogens due to the presence of the outer membrane preventing access to the peptidoglycan layer [68]. Here, a combinatorial approach, i.e., treatment of Gram-negative cells with an antibiotic to rupture the membrane, thereby allowing access to the peptidoglycan layer for lysin degradation, may be advantageous. This synergy has been observed in the treatment of *C*. *difficile* infection with the purified lysin PlyCD, where use in conjunction with vancomycin (inhibiting cell wall synthesis) was shown to have a higher inhibitory effect than either substance alone [69] (combination therapy discussed in more detail below). In the treatment of Gram-positive infections, however, it remains a powerful tool and as most phages will encode an endolysin to release progeny, a plentiful source of lytic enzymes against bacterial strains is undoubtedly available.

Endolysin research has led to the identification of useful enzymes in targeting many “drug resistant” pathogenic bacteria, as the need for alternative emerging therapies increases. Lysins have shown significant promise in murine models in the treatment of multidrug resistant bacteria, such as rescuing mice from *Acinetobacter baumanii* bacteremia [70], protecting them against systemic MRSA infection [67,71], and ex vivo treatment of *C*. *difficile* in the colon [69].

This has led to significant commercial interest in these enzymes for use in a clinical setting. Particular efforts have been made to engineer specialized lysins with increased efficacy against pathogens or displaying multifunctional activity. Due to the modular nature of phage-encoded lysins, consisting of cell wall binding domains (CWBD) and enzymatically active domains (EAD) [72], swapping of modules from other sources, or fusing of modules to other proteins can be undertaken, generating a lysin with new functional characteristics. These engineered lysins can be divided into two broad classes, chimeric lysins and artilysins. Chimeric lysins (or chimeolysins) are those which consist of domains from differing sources which have been engineered to possess improved characteristics for use. For example, ClyF active against MRSA was engineered through fusion of a staphylococcal and streptococcal lysin was found to possess improved thermostability and pH tolerance [73], fusion of the EAD from a Plyy187 (*S. aureus*) to a CWBD domain of PLyV12 (*Enterococcus*) resulted in a lysin with a wider lytic spectrum capable of infecting staphylococci, enterococci and streptococci [74]. Furthermore, it is possible to increase the lytic activity of a lysin against its desired target, as in the case of Ply187AN, a fusion of the EAD domain from Ply187 and the CWBD of LysK (Staphylococcal phage K), which shows an increased lytic capacity when compared to the original Ply187 lysin [75].

Artilysins represent a more targeted approach to engineering lysins for the purpose of overcoming the outer-membrane barrier of Gram-negative strains. It involves the fusion of an LPS-degrading peptide with the N-terminus of a lysin, puncturing the LPS layer and providing access to the peptidoglycan layer for lysin activity [76]. An example of this represents the fusion of the sheep myeloid antimicrobial peptide (SMAP29) to the KZ144 endolysin, creating Art-175 which confers the ability to puncture the outer membrane and cleave the peptidoglycan layer of *P. aeruginosa* PAO1, thereby causing a one log reduction after two minutes of treatment and a four-log reduction after 30 min [77]. Art-175 appears to have a wide lytic spectrum, capable of lysing *Klebsiella pneumoniae* [77], while also showing significant promise in lysing and disrupting persistent multidrug resistant *A*. *baumannii* strains [78].

Biofilms, microbial communities adhered to surfaces, are involved in many chronic infections and are noted for their resistance to host immune systems and medical treatments. Phages encoding depolymerases are of particular interest in the treatment of biofilm forming cultures. Due to the limited success of antibiotic therapy in treating biofilms [79], the potential to use bacteriophages or derived enzymes to treat biofilms has been under investigation. This ability is usually due to the expression of depolymerases capable of dispersing the biofilm through enzymatic digestion of extracellular polymeric substances, the main obstacle to antibiotic treatment or phage therapy [80]. The ability of phages to disrupt this biofilm is a valuable phenotype of phage therapy candidates.

The depolymerases expressed by phages digest these polymeric substances so as to obtain access to cell surface receptors [81,82], however it has been noted that the depolymerase activity alone may not be sufficient to disrupt the biofilm and the ability of the phage to amplify in the biofilm is crucial for biofilm treatment [83]. Phage-associated depolymerase activity can easily be identified in phages of interest through analysis of plaque morphology where depolymerase-expressing phages usually form a plaque surrounded by a large halo indicative of its degrading activity [84]. This phenotype has been observed for phages infecting members of several genera including *Pseudomonas* [81], *Klebsiella* [85], *Staphylococcus* [86] and *Escherichia* [87], thus representing a useful in vitro marker for phages of interest.

## 5. Overcoming Host-Encoded Phage-Resistance Mechanisms

Across phage therapy studies, the almost inevitable development of phage resistance in the targeted cells, analogous to the development of antibiotic resistance, is a primary weakness of the phage therapy concept. Therefore, the ability to overcome host resistance mechanisms represents the most valuable in vitro phenotype for a phage therapy candidate. Host resistance mechanisms fall primarily into four categories, (i) DNA degradation as provided by restriction-modification (R-M) and CRISPR-Cas systems, (ii) prevention of phage adsorption, (iii) superinfection exclusion, and (iv) abortive infection [88], along with additional hurdles to phage infection, such as the inherent resistance of biofilm communities. A summary of the following methods utilized by bacteriophages to overcome and bypass host-encoded phage resistance mechanisms is provided in Table 1.

### 5.1. DNA Degradation by R-M Systems

One of the most prevalent bacteriophage resistance mechanisms are R-M systems being present on approximately 90% of available bacterial sequences [111]. The general function of these systems is to degrade invading (unmethylated) exogenous phage DNA by an endonuclease, while providing protecting of its own DNA through methylation [111,112]. Some striking adaptations to this infection barrier are evident in the genomes of infecting phages, mostly concerning restriction inhibition. Some bacteriophages encode “orphan” methyltransferases, i.e., those lacking a restriction endonuclease partner [89]. These methylases allow self-methylation of the phage DNA, thus eliminating the ability of the host to degrade injected DNA as host encoded restriction enzymes no longer recognize the methylated restriction sites [89]. Interestingly, orphan methyltransferases in *Bacillus* phages have been observed to be multispecific, i.e., they methylate multiple recognition sites (through possession of multiple target recognition domains) thus conferring protection against a wider range of host-encoded restriction enzymes [113,114]. It should be noted that methyltransferases have been proposed to play other roles in bacteriophages. A methyltransferase in φLM21, a temperate phage of *Sinorhizobium*, has been shown to protect against restriction enzyme activity while also mimicking the activity of a host encoded regulatory methyltransferase thus suggesting a regulatory role in the phage life cycle [115]. Orphan methylases should be easily identifiable in putative therapeutic phage genomes through protein homology searches. Other less prevalent approaches involve increasing the methylase activity of hosts to encourage phage DNA methylation, typified by Ral (restriction alleviation) activity in lambda phage, which enhances the activity of host type I methyltransferases to more efficiently methylate phage DNA [90,91] or base modification of phage DNA, such as that seen in T4 which utilizes hydroxymethylcytosine (HMC) instead of cytosine which is then further modified by alpha and beta glucosylation thus rendering it impervious to many restriction enzymes through prevention of restriction site recognition [92,93].

### 5.2. DNA Degradation by CRISPR-Cas Systems

CRISPR-Cas loci encoded by many bacteria provide an adaptive response to invading bacteriophages through the incorporation of non-host DNA (protospacers), into the CRISPR array. This array acts as the memory for targeted defense against subsequent infection by protospacer-containing phages [116]. Bypass of this mechanism can be achieved in two ways, namely, mutation of protospacers and specific anti-CRISPR activity. Mutation of protospacers represents the more common strategy of CRISPR escape. High mutation rates in protospacer regions are evident during exposure to CRISPRs in the dairy bacterium *Streptococcus thermophilus* containing the corresponding spacer, here a single base change has been observed to be sufficient to return the bacterium to a phage susceptible state [94]. This phenomenon has subsequently been observed in phage infection *E. coli* [95]. Here, CRISPR spacer arrays were constructed targeting various regions of coliphages, with phage escape mutants freely isolated through standard plaque assay displaying various point mutations, deletions and insertions in the protospacer regions allowing infection of previously resistant strains [95]. The application of a high multiplicity of infection cocktail of phages should increase the likelihood of obtaining the necessary mutation in a protospacer necessary to bypass the CRISPR sequence, while application of a cocktail of phages has been seen to reduce the efficiency of a host CRISPR system to eliminate a single phage [96]. Therefore, the application of a high titer cocktail targeting a single strain should have an increased chance of success in lysing a pathogenic host.

Phage-encoded anti-CRISPR activity was first observed in *Pseudomonas aeruginosa* Mu-like phages. Here, five protein families have been identified which inhibit class 1 CRISPR type I-F systems and four protein families inhibiting the type I-E system [97,117]. These proteins are a product of distinct anti-CRISPR modules, which would be advantageous in the genomes of any phage therapy candidate [97]. The mechanisms of action of three of these proteins AcrF1, AcrF2 and AcrF3 have been elucidated, showing that they all interfere with the function of the Csy complex which facilitates the targeted recognition and cleavage of target DNA sequences in *Pseudomonas aeruginosa* [118]. Both AcrF1 and AcrF2 bind to the Csy complex of the CRISPR system, competing with crRNA for DNA binding activity [98]. AcrF3 interacts with the Cas-3 helicase nuclease protein interfering with its recruitment to the Csy complex. Homology studies have led to the identification of ten anti-CRISPR Type I-F and four anti-CRISPR type I-E genes across the Proteobacteria phylum [100]. Recently, anti-CRISPR proteins (AcrIIA) targeting Class 2 CRISPRs have been identified in *Listeria monocytogenes* prophages through identification of self-targeting spacers, i.e., a protospacer in the prophage which matches a spacer in the CRISPR array. Two phage-encoded proteins (AcrIIA1 and AcrIIA2) were found to inhibit Cas9 function allow stable co-existence of the self-targeting spacer-protospacer pair. Homologues of *acrIIA* are also present in genomes of phages infecting *Streptococcus* indicating that anti-class II CRISPR genes may be prevalent across the *Firmicutes* [99].

### 5.3. Prevention of Adsorption

Antagonistic co-evolution between phages and respective hosts is a well-documented phenomenon, defined as the reciprocal evolution of bacterial resistance and phage infectivity [119]. It has been shown for *P*. *fluorescens* SBW25 and its phage φ2 that coevolution leads to significantly increased divergence of phage genes predicted to encode the adhesion device, presumably in response to receptor changes on the host cell surface, thus preventing infection of the ancestral genotype [120]. This interaction between receptor and phage receptor binding protein is highly specific, and it has been observed in phage lambda that a combination of only four mutations allows the phage to utilize an alternative receptor [101], and a single amino acid change leads to an altered RBP specificity [40]. Lessons can be learned from this for the selection of phages for application in phage therapy. Here an attempt can be made to shift the balance in favor of bacteriophages through inclusion of multiple phages encoding a diverse range of RBPs, increasing the hurdles required for the host to acquire the necessary mutations to confer resistance against multiple receptor binding protein types. This can be achieved through large scale sequencing and phylogenetic analysis of the receptor binding protein encoding genes of candidate phages, or alternatively by individually testing phages against a panel of strains differing in cell surface carbohydrates/outer membrane molecules [102].

### 5.4. Cocktails—the Power of Many

In practice, phage therapy is typically applied in two forms. “Monophage therapy” consisting of a single phage, usually with a broad host range for application against a single species, or a phage cocktail consisting of a mixture of phages (multiphage). Phage cocktails can consist of a mixture of phages targeting a single species or a broad range of pathogenic hosts [121]. There are pros and cons associated with monophage and multiphage therapeutics. For example, a cocktail targeting a single species requires proof of an etiological agent before selection and application of the cocktail, but represents the most specific treatment available. In contrast to this, a cocktail against a range of pathogenic hosts can be applied presumptively, however, it may have a negative effect on non-target bacteria at the site of application [121]. A second advantage of cocktails is in overcoming of resistance mechanisms in the target strains, where the target would have to develop resistance to all phages in the cocktail in order to survive. For example, a study on the application of a five-different phage-containing cocktail targeting *Klebsiella pneumoniae* isolated from infected burn wounds, observed a full log reduction in target load when compared to any of the phages individually. Furthermore, the cocktail had the lowest incidence of emergence of phage resistant variants [109]. Cocktail development can be optimized to this effect. As previously mentioned, a range of different phages capable of infecting a strain and phage resistant variants can be isolated from environmental samples using the SBS method by using wild-type and phage resistant variants as hosts allowing for the formation of an effective phage cocktail [13]. In addition to this, another method first applied to *Staphylococcus aureus* strains involves the serial passaging of an isolated phage of interest against its host strain of interest followed by phage-resistant variants to enrich for broad host-range phage mutants [108]. These two approaches both lead to the development of optimized phage cocktails capable of infecting strains of interest and potential phage resistant variants. In vitro analyses of phage cocktails should be undertaken to ensure the desired phenotype. This analysis usually takes the form of time-course killing experiments comparing single phages to cocktails, while also monitoring for the emergence of resistance variants [12,110]. Another noted concept in phage cocktails is that of phage synergy where the action of one phage augments the properties of a second phage in the cocktail [110]. This has been observed under in vitro conditions, where two phages infecting *E*. *coli* exhibited a 10-fold greater ability to lyse their host when applied together compared to either phage alone. The increased capacity for lysis was theorized to be due to the stripping of colonic acid from the host cell surface by one phage (J8-85) allowing increased access to receptors on the cell surface for the other (T7-61) [110]. However, in contrast to this, the opposite effect has also been noted, the prospect of viral interference, where the possibility remains for phages to interfere with each other following co-infection [6], where co-infection with two phages may reduce observed burst sizes.

### 5.5. Combination Therapy

A further development to the power of multiple bacteriophages, is the efficacy of multiple bactericidal agents. It is theorized that resistance to bacteriophages (and antibiotics) will evolve less frequently with a combination of phage and antibiotic therapy because a strain which is resistant to a phage will be inhibited by the antibiotic and vice versa, necessitating multiple independent mutations to overcome both [103]. This phenomenon has been investigated in vitro studying *Pseudomonas fluorescens* SBW25 and its infecting phage SBW25φ2, where combined treatment with lethal concentrations of kanamycin prevented resistance development in treated samples [104]. However caution must be exercised as the opposite effect has been noted in utilizing sub-lethal concentrations of streptomycin in combination with the same phage host combination [105]. Here, an increase in resistance development was observed as well as extinction of the phage [105], clearly suggesting that combination therapy requires a high antibiotic concentration.

There is significant promise in utilizing combination therapy in the eradication of biofilms where (as mentioned above) phage-encoded, exopolyscaccharide (EPS)/capsule-degrading depolymerase activity provides access for the antibiotic to target cells. This method has shown encouraging promise in the treatment of biofilms of *E. coli* [106] and *P. aeruginosa* [107]. The previously mentioned prevention of resistant variants has also been observed in the biofilm environment where a study of combined therapy on a *K. pneumoniae* biofilm noted that combined treatment with ciproflaxin resulted in a reduced emergence of resistant variants [122]. This suggests that combination therapy has a dual benefit in biofilm treatment, increasing the capacity of antibiotics to eradicate the biofilm and preventing emergence of resistance to both components of the treatment.

Combination with antibiotic can also lead to phage-antibiotic synergy (PAS), the tendency of phages to appear more virulent in the presence of sub-lethal antibiotic concentrations first observed in uropathogenic *E*. *coli* [123]. The potential for PAS can be easily ascertained in vitro through simple one step growth curves and in vitro biofilm eradication trials [106], however due to the potential for increased resistance, caution should be observed with use of sub lethal antibiotic concentrations. In addition to this, in all antibiotic combination therapy the potential for development of antibiotic resistance in non-target cells is a considerable risk, though it should not be considered to be more probable than the risk associated with antibiotic treatment alone.

## 6. Conclusions

**Figure 1:**
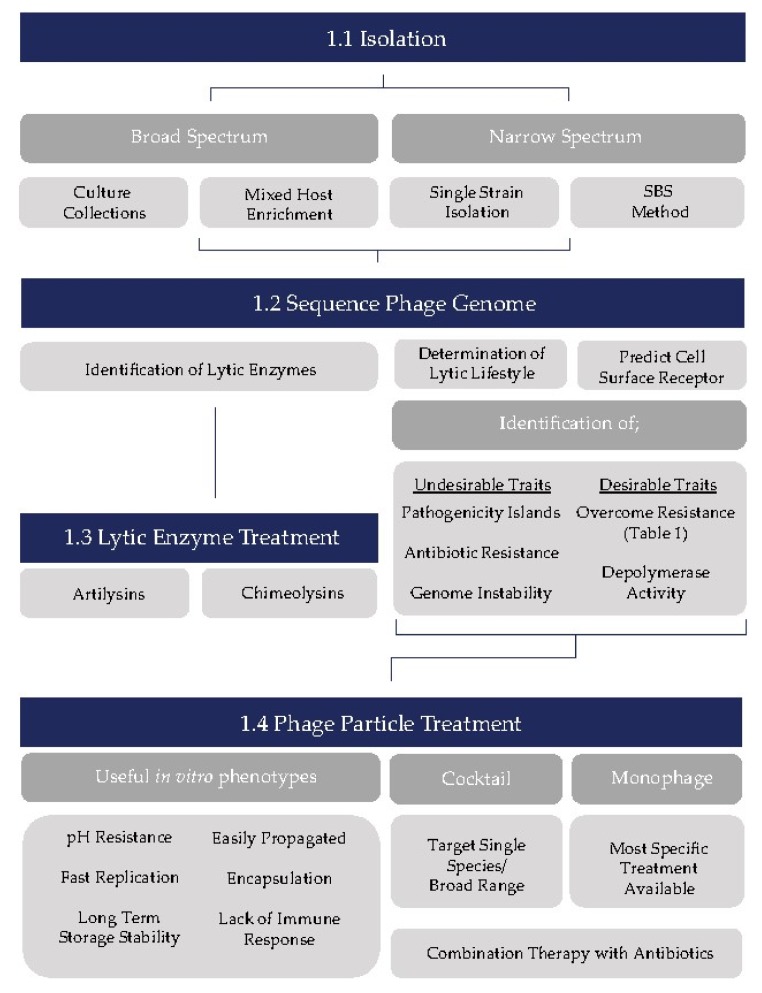
Suggested workflow for selection of phage therapy candidates, from isolation to implementation including desirable in vitro phenotypes.

Phage therapy has the potential to alleviate the ever-growing problem of antibiotic-resistance and the development of so-called “superbugs”, either as an alternative to antibiotics, or in combination with traditional antibiotic therapies to enhance their effectiveness. Despite the regulatory concerns associated with phages as therapeutic agents, phage biologists have continued in the search for phages with therapeutic potential, resulting in the isolation of countless phages that could represent an endless arsenal against a range of human and animal pathogens. Furthermore, the development of phage products such as chimeric lysins and artilysins, highlights the reservoir of antimicrobial agents that may be harnessed from phages without the need for direct application of intact phages. However, we must endeavor to overcome regulatory concerns regarding the application of intact phages, since phages are ubiquitous and are innate residents of humans. While numerous studies have been performed regarding the isolation of novel phages and their characterization, a more unified approach to the assessment of phages may be required to ensure the ultimate success of phage therapy into the future. Here, through a review of desirable phage phenotypes that phage biologists may seek out when isolating phages, we propose a workflow for selection of candidates for therapeutic purposes (Figure 1). Firstly, in isolation of phages attempts should be made to target isolation towards broad or narrow host range phages (as desired) through implementation of various isolation methods (Figure 1(1.1)). The next step in the workflow should be genome sequencing (Figure 1(1.2)) representing one of the most important steps in candidate selection for several of reasons. Firstly, it will enable identification of putative phage-encoded lytic proteins for lytic enzyme therapy (Figure 1(1.3)), and secondly it will allow assessment of the genomic characteristics (both favorable and unfavorable) of candidates for implementation in phage particle therapy. Here, we can ensure the lytic nature of candidates through identification of proteins (in particular integrase/resolvase and repressor proteins) likely to be involved in the lysogenic lifestyle preventing inefficient lysis due to lysogenic conversion. Additionally, identification of receptor binding proteins, leading to the prediction of cell surface receptors, should guide informed selection of phages for generation of a phage cocktail targeting different cell surface moieties. Genome sequencing has other added advantages in the identification of other desirable such as depolymerase activity or the ability to overcome phage resistance as well as undesirable traits such as antibiotic resistance genes, pathogenicity islands, and genome instability. Sequencing at an early stage in candidate selection is advisable, as identification of these traits would render a phage unsuitable for use in therapy, thus rendering all other characterizations redundant. Following confirmation of suitability for phage therapy (Figure 1(1.4)), phages should be assessed for suitable in vitro characteristics as discussed above before selection for use in a therapeutic setting (perhaps first by means of an animal model prior to a human clinical trial). As we face the current antibiotic crisis, this workflow should prove to be useful in the isolation and identification of phage therapy candidates, which we hope will become a viable and widespread alternative to antibiotic therapy.

## Figures and Tables

**Table 1:** Identification and application of methods to bypass phage resistance in target strains.

Resistance Mechanism	Method of Bypass	Application	Reference(s)
**Restriction modification**	Phage-encoded methyltransferases	Protein homology query for identification in candidates	[89]
	Enhancement of host methylation	Protein homology query for identification in candidates	[90,91]
	Base modification	Protein homology query for identification in candidates	[92,93]
**CRISPR**	Mutation of protospacers	High MOI to encourage mutation of protospacers	[94,95,96]
	Phage-encoded anti CRISPR systems	Protein homology query for identification in candidates	[97,98,99,100]
**Prevention of adsorption**	Mutation of receptor binding protein	High MOI to encourage mutation in RBP	[40,101]
	Selection of multiple RBP type phages	Target a diverse range of receptors on target surface	[102]
**Biofilm**	Antibiotic combination therapy	Dual-pronged inhibition of target decreasing likelihood of resistance emergence	[103,104,105,106,107]
**Emergence of phage resistant variants**	Informed cocktail development (SBS method, serial enrichment)	Selection of phages capable of infecting “future” resistant variants	[13,108]
	Selection of multiple phages infecting a single strain	Target a diverse range of receptors on target surface	[12,109,110]
